# Exploring transfer learning in chest radiographic images within the interplay between COVID-19 and diabetes

**DOI:** 10.3389/fpubh.2023.1297909

**Published:** 2023-10-18

**Authors:** Muhammad Shoaib, Nasir Sayed, Babar Shah, Tariq Hussain, Ahmad Ali AlZubi, Sufian Ahmad AlZubi, Farman Ali

**Affiliations:** ^1^Department of Computer Science, CECOS University of IT and Emerging Sciences, Peshawar, Pakistan; ^2^Department of Computer Science, Islamia College Peshawar, Peshawar, Pakistan; ^3^College of Technological Innovation, Zayed University, Dubai, United Arab Emirates; ^4^School of Computer Science and Technology, Zhejiang Gongshang University, Hangzhou, China; ^5^Department of Computer Science, Community College, King Saud University, Riyadh, Saudi Arabia; ^6^Faculty of Medicine, Jordan University of Science and Technology, Irbid, Jordan; ^7^Department of Computer Science and Engineering, School of Convergence, College of Computing and Informatics, Sungkyunkwan University, Seoul, Republic of Korea

**Keywords:** COVID-19 disease, diabetes, transfer learning, disease detection, diagnosis using deep learning

## Abstract

The intricate relationship between COVID-19 and diabetes has garnered increasing attention within the medical community. Emerging evidence suggests that individuals with diabetes may experience heightened vulnerability to COVID-19 and, in some cases, develop diabetes as a post-complication following the viral infection. Additionally, it has been observed that patients taking cough medicine containing steroids may face an elevated risk of developing diabetes, further underscoring the complex interplay between these health factors. Based on previous research, we implemented deep-learning models to diagnose the infection *via* chest x-ray images in coronavirus patients. Three Thousand (3000) x-rays of the chest are collected through freely available resources. A council-certified radiologist discovered images demonstrating the presence of COVID-19 disease. Inception-v3, ShuffleNet, Inception-ResNet-v2, and NASNet-Large, four standard convoluted neural networks, were trained by applying transfer learning on 2,440 chest x-rays from the dataset for examining COVID-19 disease in the pulmonary radiographic images examined. The results depicted a sensitivity rate of 98 % (98%) and a specificity rate of almost nightly percent (90%) while testing those models with the remaining 2080 images. In addition to the ratios of model sensitivity and specificity, in the receptor operating characteristics (ROC) graph, we have visually shown the precision vs. recall curve, the confusion metrics of each classification model, and a detailed quantitative analysis for COVID-19 detection. An automatic approach is also implemented to reconstruct the thermal maps and overlay them on the lung areas that might be affected by COVID-19. The same was proven true when interpreted by our accredited radiologist. Although the findings are encouraging, more research on a broader range of COVID-19 images must be carried out to achieve higher accuracy values. The data collection, concept implementations (in MATLAB 2021a), and assessments are accessible to the testing group.

## 1. Introduction

The intersection of COVID-19 and diabetes represents a multifaceted area of concern in contemporary healthcare. Diabetes, a chronic metabolic disorder characterized by high blood sugar levels, has emerged as a significant risk factor for severe COVID-19 outcomes (1). Emerging research has illuminated a complex relationship, revealing that individuals with diabetes are more susceptible to severe COVID-19 complications and adverse consequences, such as hospitalization and mortality. This heightened vulnerability is thought to be linked to the dysregulation of the immune system and impaired inflammatory response often associated with diabetes. The COVID-19 pandemic has raised concerns about the potential development of new-onset diabetes in individuals infected with the virus. Several studies have reported cases of acute or transient diabetes occurring in COVID-19 patients with no prior history of the condition (2). While the mechanisms behind this phenomenon remain under investigation, it is believed that the virus may directly impact pancreatic function or trigger an autoimmune response, resulting in temporary or long-term diabetes.

Beyond the realm of COVID-19, another facet of the diabetes narrative emerges in the context of cough medicines containing steroids (3). Steroids are known to influence blood sugar levels, and patients who require these medications to manage respiratory conditions such as asthma or chronic obstructive pulmonary disease (COPD) can face an increased risk of developing steroid-induced diabetes (4). Physicians must exercise caution and closely monitor patients with pre-existing diabetes or those at risk of developing the condition when prescribing such medications (5). However, the positive RT-PCR rate for the sample of nose swab samples is expected to be between 30 % and 60 % (30–60%) (6), resulting in undiagnosed patients that can infect a considerable amount of those people who are young and healthy (7). The daily use of the X-ray imaging method for diagnosing pneumonia is fast and straightforward. COVID-19 may be diagnosed with elevated Sensitivity using chest CT scans (8, 9). The images of chest X-ray images reveal sensory cues linked to the coronavirus (10). Multipolar involvement and opacities in the peripheral airspace are seen in chest imaging studies. Frosted glass (57 percent) and mixed mitigation (29 percent) are the most often mentioned opacities (11). A frosted glass pattern can be seen in areas bordering the pulmonary vessels at the start of COVID-19, which is challenging to determine visually (12). COVID-19 has also been linked to airspace opacities that are uneven or diffusely asymmetric (13). Expert radiologists are the only ones that can interpret these apparent anomalies. Automatic methods to detect these subtle anomalies may facilitate the diagnostic process and increase the early detection rate considerably, given many suspicious individuals and the small number of qualified radiologists.

The COVID-19 outbreak, generally regarded as the third coronavirus outbreak, affected over 209 countries, one of which was Pakistan. The COVID-19 epidemic, which first broke out in China, severely impacted the countries that border Pakistan, including China. China was also the country where the epidemic began. Italy had the highest mortality rate of COVID-19 in the western region, while Iran had the second-highest mortality rate in the northern part (14). Italy was also the country with the highest incidence of COVID-19. The COVID-19 virus was identified in Pakistan’s first patient on February 26, 2020, by the Ministry of Health under the administration of the Pakistani government. The patient’s location was determined to be in Karachi, which is the largest city and provincial capital of Sindh. On the same day, a second confirmed case was found in Islamabad, which is the location of the Federal Ministry of Health of Pakistan (15, 16). Within fifteen days, the total number of confirmed cases in the province of Sindh reached twenty (17) out of a total of 471 suspected cases. This was followed by the region of Gilgit Baltistan, which had the second-highest number of confirmed disease cases. All of the people whose cases have been verified have a history of having recently traveled from London, Tehran, or Syria. These reports are currently rising rapidly, which paints an even more dire picture of the situation than was previously presented.

The relationship between COVID-19 and diabetes is complex and multifaceted. People with diabetes are at increased risk of developing severe COVID-19, and COVID-19 can also worsen diabetes management. This is due to a variety of mechanisms, including increased ACE2 expression, insulin resistance, chronic inflammation, and cytokine storms. COVID-19 can also trigger new-onset diabetes in some people, and pregnant women with diabetes are at even higher risk of developing severe COVID-19. People with diabetes who have had COVID-19 may be more likely to experience long-term effects of the virus. It is important for people with diabetes to take steps to protect themselves from COVID-19 and to manage their diabetes carefully.

**Figure 1:**
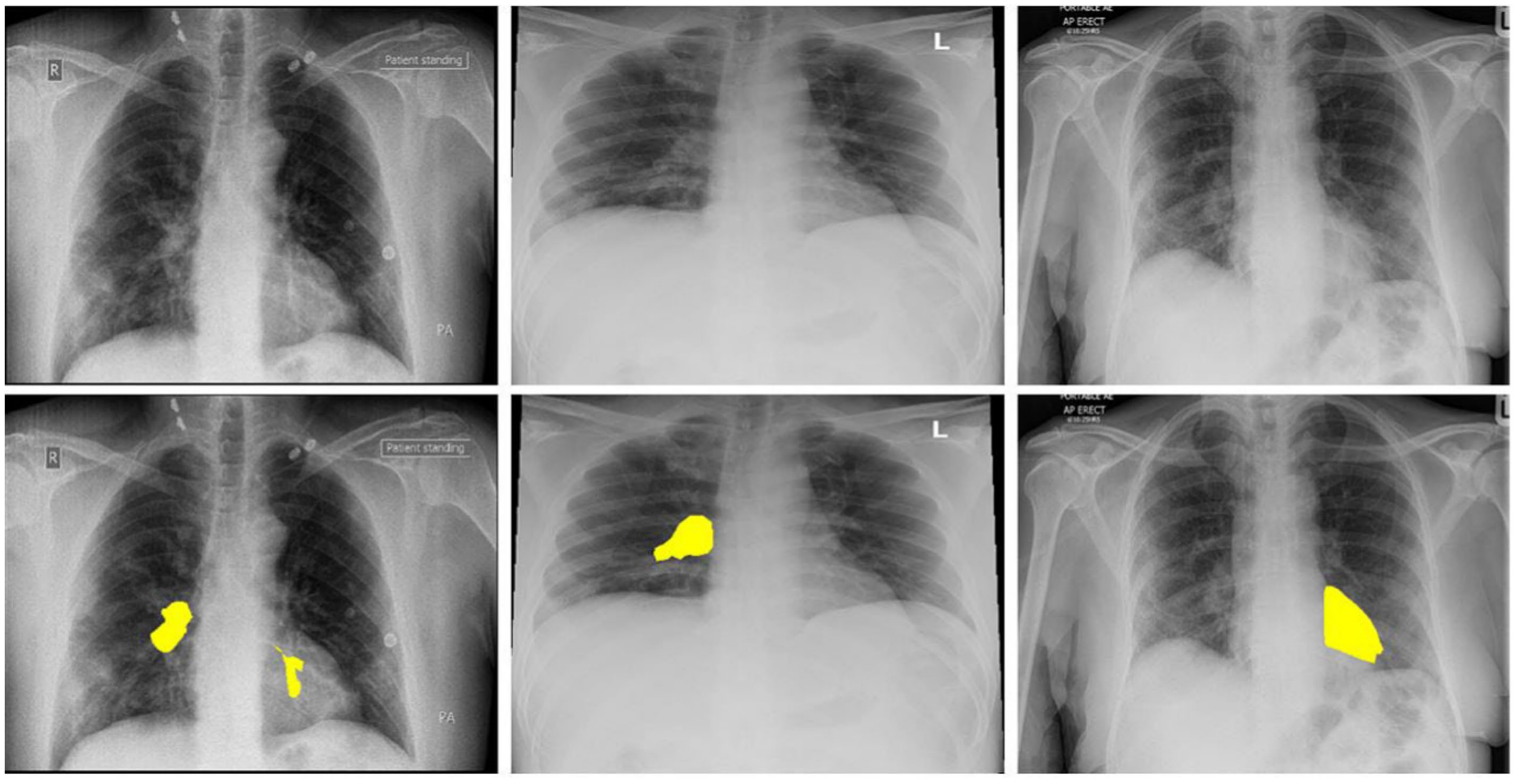
The above images are the 3 COVID-19 imageries samples and equivalent marked infected areas by our radiologist.

Artificial intelligence (AI) and deep learning solutions can be very effective in addressing these issues (18). Detailed reports documenting solutions for automated identification of coronavirus from chest X-ray images are not accessible at this time due to a shortage of public images of COVID-19 patients. A limited collection of data on images was recently obtained. This enables the researchers to create a machine-learning model that can diagnose COVID-19 *via* X-ray images of the chest (19). All of these photos were taken from research papers that reported on COVID-19 X-ray and C-Cmometric picture results. We re-labeled these X-ray images with a trained radiologist’s aid, keeping just the simple sign of the coronavirus. Our radiologist defines these labeled X-ray images. Figure 1 shows three samples of images with their labeled regions. Then, as negative samples for COVID-19 identification, we used a subsection of medical images from the ChexPert dataset (20). The consolidated dataset (called COVID-Xray-3k) contains approximately 3,000 thoracic X-ray pictures, split into 2,100 training and 900 research samples.

In order to develop a reliable deep learning based COVID-19 detection model, the size of the dataset plays a significant role, and it has a direct impact on model generalization. For augmentation of the dataset, various image processing techniques were applied, including sharpening, blurring, contrast adjustment, intensity modification in the red, green, and blue channels, shearing effects, and rotation. The augmentation process enlarges the dataset size; the model receives a lot of COVID-19 image data to learn and recognize a broader spectrum of patterns and variations in chest X-ray images. Furthermore, data augmentation also contributes to clinical relevance. In medical imaging, patient diversity and variations in image quality are prevalent. Augmenting the dataset with various transformations helps the model better account for these real-world complexities. For instance, rotation and shearing effects mimic potential variations in patient positioning during imaging procedures, while adjustments in image intensity account for differences in equipment settings and patient characteristics.

COVID-19 was predicted from thoracic X-ray images using a machine learning framework. We went in-depth on an end-to-end learning system that explicitly forecasts the raw images of COVID-19 diseases without the need to extract characteristics, in contrast to traditional approaches to the classification of images, also called medical image classification that adopts a two-step process (extraction of artisanal features – recognition). In recent years, studies (17, 21–23) have shown that in-depth learning-based models, i.e., Convolutive Neural Networks (CNN), surpass the traditional AI techniques in the domain of computer vision and medical image processing. Thus, these models are being applied to analyze problems ranging from classification, segmentation, and facial recognition to achieving high resolution and enhancing the images.

We use the COVID-Xray-3k dataset to create four standard convoluted networks that have shown promise in many tasks over the last few years and study their success in COVID-19 detection. The training steps could not be done from scratch for these networks since there are just a few widely accessible X-ray photos for the COVID-19 range. To resolve the issue of COVID-19 images absence, in this study, two techniques were used: We used the increased data to produce a modified version of COVID-19 pictures (such as spinning, a minor rotation, and inserting a small number of distortions) for increasing the images in the dataset by a factor of five. We optimize the former layer of a variant of the models on ImageNet rather than driving them from scratch. In this, the model can be built up with fewer tagged samples. These samples can be separated from each class in this manner. The two techniques described above aided in forming these networks using the accessible images and achieved good results on the test range of 30 0 0 images. We also quantify the trust interval of performance measurements since, in the COVID-19 class, the number of samples is small. The curves of receiver operating characteristics (ROC) and the region under or below the curve (AUC) of the proposed classification models are provided to summarize their output. Below are the article’s significant contributions:

- To diagnose COVID-19 from pulmonary radiographs in the form of images, we prepared a data set of three thousand images with binary tags. For the testing group, this data collection should be used as a tool. A board-certified radiologist marks the pictures in the COVID-19 class. Only those images that were used for research purposes got clear and visible signs or marks.
- Using this dataset, we qualified four successful deep learning models and tested their output on a test collection of three thousand X-ray images. The top model that performed had a sensitivity rate of ninety-eight (98%) percent and a precision rate of ninety-two (92%) percent.
- We presented an experimental study based on the systematics of these models. This experimental study was a performance comparison between several CNN models where the performance evaluation is performed using the accuracy, F1-score, and the curve of ROC and AUC. The expected probability distribution for three classes is performed using the pie chart. Using a specific visualization method, we generated thermal maps of the most probable areas infected by COVID-19.
- This study leverages state-of-the-art CNN transfer learning models to design a sophisticated system capable of achieving heightened accuracy in the detection of two distinct categories: COVID-19 without comorbidity and COVID-19 with diabetes. Additionally, the system excels in precisely localizing the affected regions within X-ray images, providing valuable insights for medical diagnosis.

The objective of this study is to develop a deep-learning model for COVID-19 patient prediction. We are also working to identify clinical data characteristics that may influence the COVID-19 outcome prediction. With the number of coronavirus-positive cases increasing daily, testing is impossible due to time and cost constraints. In recent years, machine learning in the medical field has become extremely reliable. The currently available models are developed on a relatively modest dataset, and the vast majority of the researchers have made use of a dataset that was not annotated by subject matter specialists (radiologists). The majority of the work that has been done in the field of machine learning has been accomplished through the use of hand-crafted methods and traditional approaches. The traditional methods have several performance flaws. To save human lives, a reliable and effective COVID detection system is required.

In the notice, although the results of this work are promising considering the volume of data tagged, they are only tentative, and a more definitive conclusion would take more studies in a broader dataset of COVID-19-labeled X-ray pictures. This study should be deemed as a starting point for potential research and comparisons.

The following is the outline for the remainder of this paper. Section 2 summarizes the prepared COVID-Xray-3k dataset. The proposed general structure has been explained in section 3. Experimental studies and parallels with previous work are presented in section 4. Lastly, the essay is closed in the 5th section.

## 2. The Xray-3k COVID dataset

The thoracic X-ray image from two datasets was combined to generate Covid X-ray 3,000 dataset images comprising 2,100 images for training and 900 images for testing purposes. The newly issued Covid-Chestxray-Dataset, which includes collecting X-ray images of articles published on the topic of coronavirus, was compiled by Cohen et al. https://github.com/ieee8023/ covid-chest-ray-dataset (2020). The dataset uses a mixed combination of CT scans with the images of chest X-rays. The dimension of CT images is 512x512x28 with a bit depth of 16 bits, and the file format is volumetric DICOM; similarly, the X-ray image size is 1024x1024x1 with a bit depth of 12 and 16 bits DICOM images. The images generated until May 3, 2020, contained two hundred and fifty X-ray images of corona-infected patients, with two hundred and three images corresponding to anteroposterior views. This data collection is continually modified according to the description. It also includes information about each patient, such as gender and age. Collecting images from both the CT scans and Xray diverse sources is a strategy employed in our study to enhance the comprehensiveness and robustness of our COVID-19 detection model. While domain adaptation and shifts pose challenges, our rigorous approach to data preprocessing, feature extraction, and model calibration is designed to mitigate these effects. By addressing these challenges head-on, we strengthen our model’s reliability and real-world applicability, ultimately advancing the field of medical image analysis for COVID-19 diagnosis. This dataset provided us with all of our COVID-19 images. According to our accredited radiologist’s recommendation, only anteroposterior X-ray samples are held to forecast COVID-19, as the previous samples were not considered appropriate for that reason. A qualified radiologist analyzed the anteroposterior images, and those lacking even the tiniest X-ray symbol of coronavirus were omitted from the data collection. 19 of the 203 COVID-19 indoor-outdoor X-ray images were discarded, leaving 184 for our radiologist to examine (which depicted clear indications of COVID-19). As a result, we would include a more accurately labeled data collection for the world. Among these images, 100 images per class are used for the testing (to achieve the highest value of confidence interval), while the remaining images are used as the training set. As previously mentioned, the data improvement is added to the learning kit to escalate the number of COVID-19 samples to 420.

Both patient X-ray images are transmitted only on one of the training courses, as we have ensured. Our radiologist highlighted the areas of clear Covid-19 signs due to the low number of images with no coronavirus collected on the dataset (20). This dataset includes 0.22 million images and three hundred and sixteen (224,316) chest X-ray images of sixty-five thousand two hundred and forty (65,240) patients. It is marked with the indication of 14 subcategories (non-finding, edema, pneumonia, etc.). We used only images from a single subcategory for non-COVID samples from the learning package, which consisted of seven hundred (700) pictures from the non-research class and one hundred (100) image from every other thirteen (13) subclasses, totaling two hundred (200) non-COVID images. We picked 1,700 images from the unsearched division.

**Figure 2:**
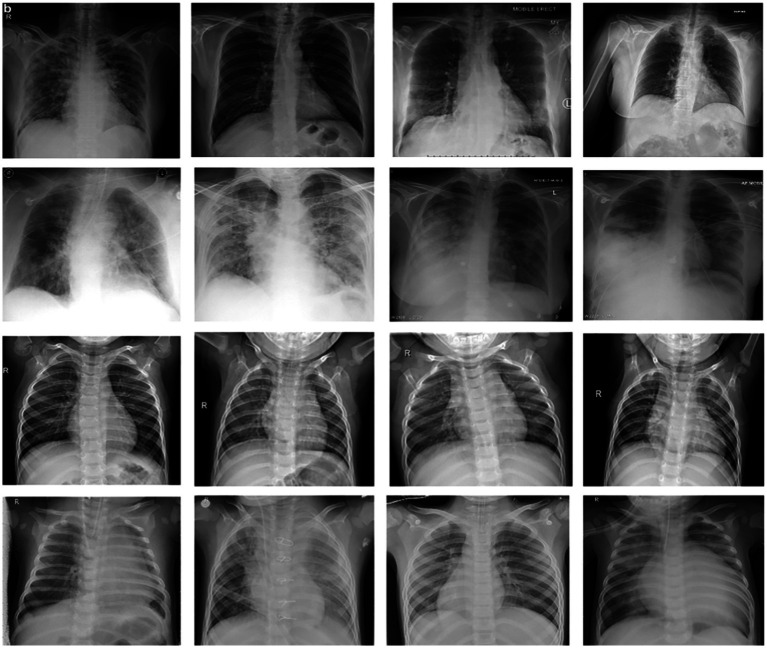
Sample of images from COVID-Xray-3k Dataset. First row corresponds to images with COVID-19. The second row corresponds to four sample images diagnosed with no COVID-19 infection from ChexPert, belonging to the no-finding category. The third and fourth row corresponds to images with eight samples belonging to all other subdomains in ChexPert.

We picked approximately a hundred (100) images from each of the other thirteen (13) subclasses in different sub-files for non-COVID samples from the research dataset, totaling 30,000 images. Table 1 shows the exact amount of X-ray images from each class used for preparation and research. Figure 2 displays 16 photos from the COVID-Xray-3k dataset, comprising four Coronavirus images (1st row), four regular ChexPert images (2nd row), and eight images of one of the 13 ChexPert images (3rd and 4th row).

**Table 1:** Each category has no. of images in the Xray-3k COVID dataset.

Dataset Split	Non-COVID images	COVID-19 images	COVID-19 + Dibetic images
No. of training sets	700	700	700
No. of test sets	300	300	300

It should be remembered that the resolution of the photos in this data collection varies significantly. Low-resolution COVID-19 images (less than 400 × 400 pixels) and high-resolution COVID-19 images (over 1900 × 1,400 pixels). This is a plus for models who can reach a reasonable precision level on this data collection, considering the variable image resolution and imaging technique. Although gathering all the photos in a highly controlled system, we desired to get ultra-sharp images with very high-resolution images; it is not always possible. As machine learning advances, more focus is put on the models and frames that will perform. On low-quality, small-scale tagged data sets, it performs reasonably well. Furthermore, the original vendor collects COVID-19 class images from various sites, showing dynamic variations (and even from ChexPert). However, the whole dataset is optimized to the same distribution in the testing phase to make the model less vulnerable to this.

Pursuing higher accuracy in COVID-19 diagnosis through deep learning models is challenging, and it necessitates an ongoing effort to access diverse and extensive datasets. To achieve this, researchers can explore several avenues. Public medical databases, such as the National Institutes of Health (NIH) Chest X-ray Dataset and the COVID-19 Image Data Collection, offer open-access repositories of radiographic images that can significantly augment existing datasets. Collaboration with medical institutions and hospitals can provide access to real-world patient data, capturing different COVID-19 manifestations and stages.

## 3. The proposed framework

Transfer learning was used to modify four deep neural networks and pre-trained images of the COVID-Xray-3k Dataset to solve the small data sizes. The choice of selection of the state-of-the-art transfer learning models in our study for COVID-19 detection using the x-ray images was based on their diverse architectural characteristics and well-established performance in image analysis tasks. These selected models are well known for their robustness, efficiency, and ability to transfer knowledge from large-scale datasets. This deliberate model selection aimed to comprehensively evaluate their suitability for COVID-19 detection and contribute to the advancement of medical image analysis.

### 3.1. Method of transfer learning

In this method, a model that has been educated on one task is reassigned to a similar task and is expected to respond to the new task. For, consider using an ImageNet model used to classify images (which includes billions of labeled images/pictures) to kickstart learning that will also be task-specific. This is used to detect COVID-19 on minor data collection. Transfer learning is most useful for those projects that require only a little effort to build models from the scrape, such as medical-based image recognition for evolving chronic diseases.

This is true, particularly for deep neural network-based models, which have many parameters to learn. In transfer learning, the setting of the model has better initial values, which needs a few minor improvements to make them more structured for the new mission. For each task, the pre-trained model is used in one of two ways. The first method is viewed as a model that extracts the characteristics, i.e., an extractor. In the second method, the model is trained to classify a classifier.

Another method involves purifying the whole network, or a subset of it, for the current mission. We simplify the end layer of complicated neural networks because the number of samples in the COVID-19 segment is relatively less. Consequently, the weights and biases of pre-trained CNN models are used to be a starting point for the proposed study, which are revised throughout the learning process. We use previously trained models as a characteristic extractor. ResNet-18 (24), ResNet-50 (25), Inception-ResNet-v2 (26), and NASNet-Large are four standard pre-formed models that we evaluate (27). The following segment gives a brief description of the models’ design and their implementation to recognize coronavirus.

### 3.2. Inception-v3 and ShuffleNet based COVID-19 detection

The pre-designed Inception-v3 model, formed on the ImageNet dataset, is one of the models implemented in our research. Inception-v3 is one of the most common CNN architectures, and it won the 2015 ImageNet contest. It offers a more effortless gradient flow for more effective training. The implementation of an identity shortcut link that misses/skips one or more than one layer is at the heart of Inception-v3. This will enable the network to have a clear route to the network’s first layers, rendering gradient changes far simpler for these layers. Supplementary Figure S1 depicts the Inception-v3 model’s general theory scheme and its application to COVID-19 identification. The Inception-v2 design is similar to Inception-v3 but with a number of layers than the Inception-v3. The structure design of ShuffleNet CNN features learning and classification can be seen in Supplementary Figure S2. Supplementary Figures S4–S7 illustrates the probabilities estimated by the various CNN models when applied to the testing samples. This graphical representation provides valuable insights into the model’s confidence scores and its decision-making process.

### 3.3. The inception-ResNet-v2 for COVID-19 detection

The Inception-ResNet-v2 is a small CNN model that obtains accuracy up to the AlexNet level (28) with 50 times more minor settings. Using these techniques, the biographers compressed Inception-ResNet-v2 to a smaller amount, i.e., smaller than 0.5 MB, making it prevalent for applications requiring lightweight models. They substitute one layer 1 × 1 that “tightens” the data entering the vertical dimension, followed by the sign of two parallel convoluted layers 1 × 1 and 3 × 3 that “extend” the data’s depth again. Inception-ResNet-v2 services three effective strategies: replacing 3 × 3 filters with 1 × 1 filters, growing the number of input channels to 3 × 3 filters, and subsampling late in the network to ensure massive activation maps for convolution layers.

### 3.4. COVID-19 detection using NASNet-large

Another architecture introduced by (29) is the Neural Architectural Seach Convolutional Network (NASNet-Large), which won the ImageNet 2017 competition. Each layer in NASNet-Large receives additional entries from all preceding layers and transmits its function cards to all succeeding layers. Each layer gets all of the previous layers’ accumulated information. The network can be thinner and more lightweight because every layer receives maps for every layer. Supplementary Figure S3 depicts the architecture of the NASNet-Large example.

### 3.5. Model training

The cross-entropy loss function, whose goal is to decrease the change between expected probability scores and field truth probabilities, is used to train all models.


(1) $$L_{CE}=-\overset{N}{\underset{i=1}{\sum }}p_{i}\log\;q_{i}\;$$

Where *pi* denotes ground truth, whereas *qi* denotes predicted probabilities for every image. A stochastic gradient descent algorithm can then be used to minimize this loss function (and its variations). We tried to improve the loss feature by including regularization, but the resulting model did not improve.

## 4. Results

### 4.1. Hyper-parameters model

**Figure 3:**
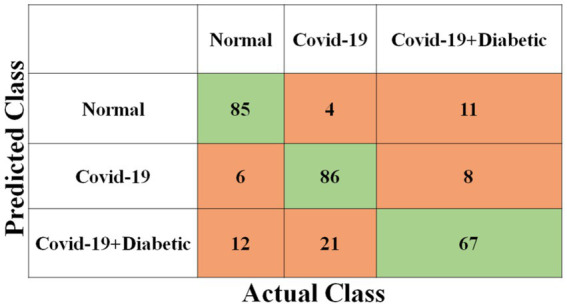
Shows the proposed Inception-v3 model’s confusion matrix.

**Figure 4:**
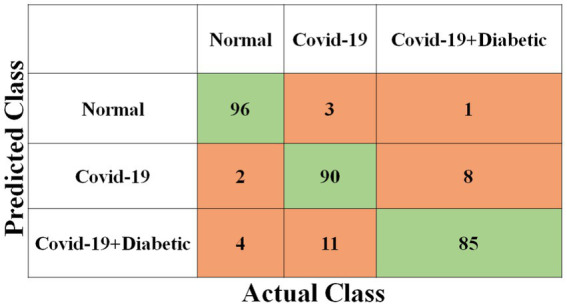
Shows the proposed ShuffleNet model’s confusion matrix.

**Figure 5:**
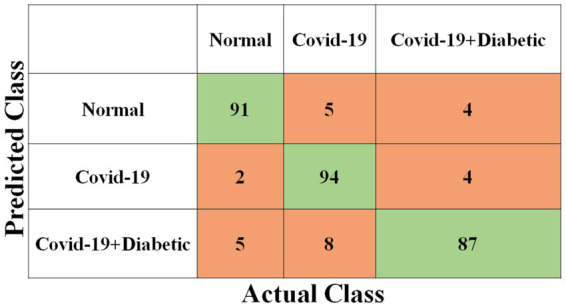
Shows the proposed Inception-ResNet-v2 CNN model confusion matrix.

**Figure 6:**
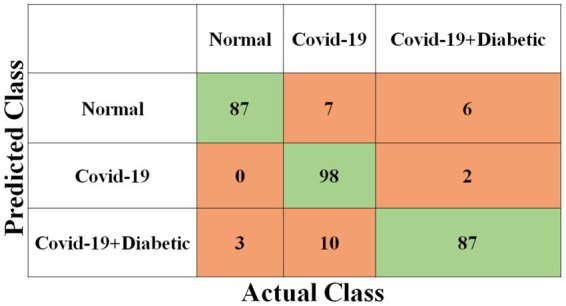
Shows the proposed NASNet-Large CNN model confusion matrix.

Each model has been trained with 100 Epochs. The loss function is optimized with the use of an ADAM optimizer having a learning rate of 0.0001. This optimizer has a size of 20. Since these models are typically created with a detailed image resolution, all the images are under 224*224 before being submitted to the neural network. All the experimental tasks are performed using the MATLAB deep learning framework. The confusion matrices for the four classification models, each tasked with classifying three distinct classes, are presented in Figures 3–6. These visual representations provide a comprehensive view of the models’ performance in categorizing instances into “Normal,” “Covid-19,” and “Covid-19 + Diabetic” classes.

The Supplementary Table S1 displays the hyperparameters used, their corresponding values or methods, and the optimal selections during the training of four transfer learning CNN models. The Inception-v3 CNN achieved an average accuracy of 79.62%, the Figure 3 displays correctly predicted samples in green, while incorrectly predicted samples are shown in red. Similarly, Figures 4–6 display the confusion matrices obtained when validating the test set with ShuffleNet, Inception-ResNet-v2, and NASNet-Large, each surpassing accuracy rates of 90.33, 90.67, and 90.67%, respectively. These remarkable accuracies underscore the effectiveness of the chosen optimal hyperparameters.

### 4.2. Evaluation metrics

Different metrics, including classification precision, Sensitivity, specificity, accuracy, and F1 ranking, may be used to evaluate classification models’ success. Due to the unbalanced nature of the current test dataset (80 coronavirus infectious images vs. 2000 non-coronavirus infectious images), sensitivity and specificity are two critical indicators to report model performance:


(2) $$\mathrm{Sensitivity}=\frac{\begin{array}{l} \mathrm{The number of images}\\ \mathrm{correctly predicts COVID}19 \end{array}}{\;\mathrm{The total COVID}19\;\mathrm{images}}$$

(3) $$\mathrm{Specificity}=\frac{\begin{array}{l} \mathrm{The total number of images}\\ \mathrm{correctly predicted}\;\mathrm{as}\;\mathrm{NonCOVID} \end{array}}{\mathrm{The total number of NonCOVID images}}\;$$

### 4.3. The predicted scores of models

We are based on four standard convoluted networks, as previously stated. All these models generate a probability score for every X-ray image. It also increases the probability factor of the disease being identified as COVID-19. We may develop a binary mark to indicate whether the image is COVID-19 or not. We can get this by the comparison of the binary Mars with a cut-off threshold. A perfect model can detect/predict the chance for every COVID-19 sample, which is found to be close to 1. Like this, an ideal model can predict the possibility of every non-COVID sample being close to 0. Tables 2–5 Present the Sensitivity and Specificity Achieved by Four CNN Models for the Detection of COVID-19 with Diabetes. Table 2 presents the sensitivity and specificity achieved by the Inception-v3 model across various threshold values. Meanwhile, Tables 3–5 provide sensitivity and specificity values for the ShuffleNet, Inception-ResNet-v2, and NASNet-Large models, respectively.

**Table 2:** The results of the Inception-v3 model in the form of sensitivity and specificity rates.

Sensitivity	Specificity	Threshold
100%	73.4%	0.19
99%	92.7%	0.18
96%	94.4%	0.22
93%	96.8%	0.23
87%	98.0%	0.31

**Table 3:** The results of the ShuffleNet model in the form of sensitivity and specificity rates.

Sensitivity	Specificity	Threshold
100%	79.2%	0.17
97%	90.2%	0.24
95%	95.3%	0.21
92%	98.5%	0.29
87%	98.4%	0.36

**Table 4:** The results of the Inception-ResNet-v2 model in the form of sensitivity and specificity rates.

Threshold	Sensitivity	Specificity
0.32	98%	91.2%
0.19	99%	90.4%
0.4	97%	95.8%
0.39	93%	98.2%
0.8	88%	99.7%

**Table 5:** The results of the NASNet-Large model in the form of sensitivity and specificity rates.

Threshold	Sensitivity	Specificity
0.16	97%	77.3%
0.22	94%	89.8%
0.29	92%	96.4%
0.38	81%	99.8%

Supplementary Figures S4–S7 display the model’s distributions of expected likelihood scores for the test set photos, respectively. We include the probability distribution of the expected three categories: COVID-19, Normal, and other diseases. Our study’s non-COVID grouping consists of both standard cases and other forms of diseases. As can be said, non-COVID X-ray images of different types of infections have significantly better ratings than non-COVID examples without other types of diseases. The infected images of COVID-19 may have somewhat higher odds than non-COVID images, which is promising. We can see that Inception-ResNet-v2 is better at work than the other models. Table 6 provides a comprehensive overview of the class-specific performance metrics, and the average performance of four state-of-the-art CNN models used for chest radiography detection. The models were evaluated across three distinct classes: “Normal,” “Covid-19,” and “Covid-19 + Diabetic.” The metrics examined include Accuracy, Precision, Recall, and F1-score, offering valuable insights into the models’ capabilities for each class. Additionally, the table presents an “Average” row, summarizing the collective performance of these models across all classes. These metrics serve as a vital reference point for evaluating the models’ effectiveness in detecting and distinguishing between different chest radiography categories.

**Table 6:** Class-specific Performance metrics and average performance of state-of-the-art CNN models for chest radiography detection.

Model	Class	Accuracy	Precision	Recall	F1-score
Inception-v3	Normal	85.85%	88.57%	89.47%	89.01%
	Covid-19	86.00%	86.00%	93.33%	89.33%
	Covid-19 + Diabetic	67.00%	67.00%	72.00%	69.33%
	Average	79.62%	80.52%	84.97%	82.72%
ShuffleNet	Normal	96.00%	96.00%	96.00%	96.00%
	Covid-19	90.00%	90.00%	92.00%	91.00%
	Covid-19 + Diabetic	85.00%	85.00%	87.00%	86.00%
	Average	90.33%	90.33%	91.67%	90.67%
Inception-ResNet-v2	Normal	91.00%	93.57%	95.56%	94.51%
	Covid-19	94.00%	94.00%	96.00%	95.00%
	Covid-19 + Diabetic	87.00%	87.00%	89.00%	88.00%
	Average	90.67%	91.52%	93.52%	92.52%
NASNet-Large	Normal	87.00%	90.55%	91.89%	91.21%
	Covid-19	98.00%	98.00%	98.00%	98.00%
	Covid-19 + Diabetic	87.00%	87.00%	89.00%	88.00%
	Average	90.67%	91.85%	92.96%	92.41%

### 4.4. The sensitivity and specificity of four different models

Every model generates a probability score that indicates the likelihood of the image, i.e., the idea being COVID-19. These scores are then compared to a criterion to determine whether or not the picture is COVID-19. The value of the Sensitivity of all models and the importance of the specificity of all models were calculated using predicted labels. Tables 2–5 demonstrate sensitivity rates and specificity rates for various levels utilizing the four models. It can be shown that both of these models provide positive outcomes, with the strongest one achieving a sensitivity of 95% (95%) and specificity of 91% (91.06%). Inception-ResNet-v2 and Inception-v3 outperform the other models by a small margin.

The Inception-ResNet-v2 has the high sensitivity (98%) and specificity (91.2%) rates demonstrated by our top-performing model, which holds substantial clinical significance. These performances reflect the model capability of accurately detecting COVID-19 cases and reducing the inaccurate diagnosis. The higher accuracy of the model assists in early disease diagnosis and treatment plans, making it a vital tool for radiologists and pulmonologists. Moreover, the model flexibility for different clinical scenarios is necessary, as indicated by various threshold options, to enhance its practical use in real-world applications, where balancing sensitivity and specificity is crucial for effective COVID-19 diagnosis.

### 4.5. The reliability of the model with a few cases of COVID-19

It should be mentioned whether the sensitivity and specificity rates shown earlier can be or cannot be accurate because there was a minimal amount of accurately annotated COVID-19 X-ray images by the experts who are available to date besides the fact that the COVID-19 dataset consists of several hundred X-ray samples. More studies on more test samples are required to get a more accurate estimate. To see the potential range of these values in every class, we will measure the confidence interval at 95% of sensitivity and specificity rates recorded here. The accuracy rate trust interval can be determined as follows:


(4) $$r=\sqrt[{z}]{\frac{accuracy\left(1-accuracy\right)}{N}}$$

Where z is the confidence interval’s degree of significance, accuracy is the approximate accuracy (in our case, sensitivity rates and specificity rates), and N is the total number of samples. In this case, we used a 95 percent trust interval, which corresponds to a z-value of 1.96. Since a responsive model is critical for the COVID-19 diagnosis, we select a cut-off threshold for each model that fits a sensitivity rate of 98 % (98%) and can also evaluate their specificity values. Supplementary Table S2 shows how these four models performed throughout the test range. Since we have around three thousand samples for this class, the confidence interval for specificity values is minimal (around 1%). In contrast, the sensitivity rate has a somewhat higher confidence interval (about 2.7%) due to the smaller number of samples. The performance comparison is presented in Table 7, incorporating the latest advancements from state-of-the-art research.

**Table 7:** Comparison of the proposed model with existing state-of-the-art methods.

Model	Accuracy	F-Measure
CovidxNet-CT (30)	85%	86.06%
Optimized Resnet 101 (31)	95%	93.32%
UNet+ ResNet (32)	94%	92.3%
EfficientNet+SCO (32)	85%	87.66
Proposed Model (33)	96%	96.9%

### 4.6. The operating characteristics curve (ROC)

**Figure 7:**
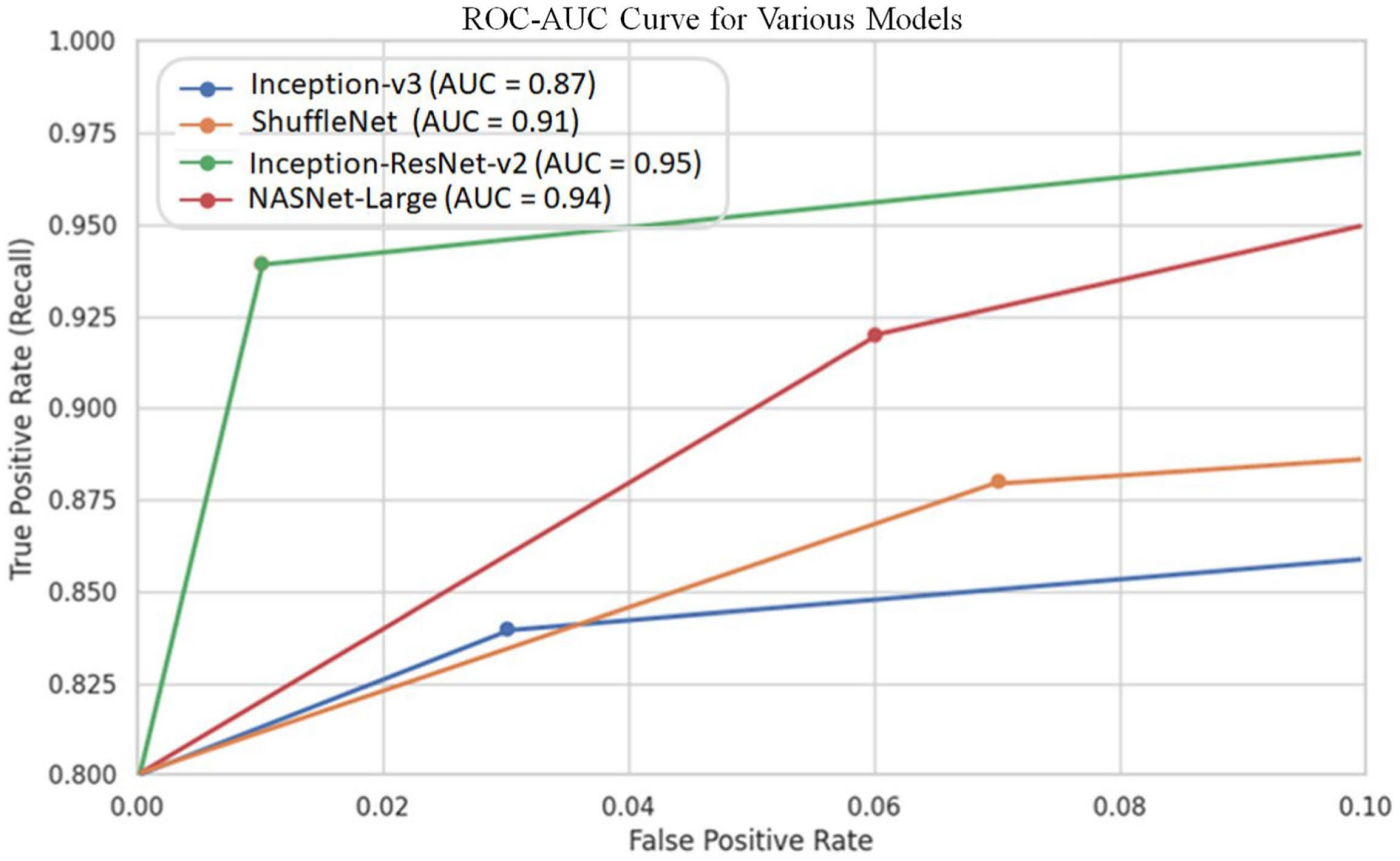
Shows the precision-recall curves of 4 CNN architectures for COVID-19 detection.

Since cut-off limits vary, it is challenging to equate various models. We ought to test all potential threshold values to see how these models compare overall. The precision-recall curve is one way to do this. Recall or Sensitivity is the Ratio of true positives to total (actual) positives in the data. Recall and Sensitivity are one and the same. Whereas the accuracy is calculated using the accurately detected +ve images and the total number of +ve images in the test set using the ROC curve. Figure 7 depicts the curve created using the precision and recall values of the proposed CNN models. The ROC curve is plotted by taking the precision values on the y-axis and recall values on the x-axis of the 2D line plot. Supplementary Figure S8 shows the ROC curves of these four models. Both versions work equally according to AUC.

It should be noted that the AUC might not be a suitable predictor of model success for very unbalanced test sets (because it can be very high) and that examining the medium accuracy curve and precision and recall may be a safer option in this case. For the sake of completeness, we have included all curves here. The confusion matrices of the two highest-performing CNN models, Inception-v3 and Inception-ResNet-v2, on a test set of 2080 Xrays can be observed in Figures 3, 5. These matrices provide an exact count of suitable samples, i.e., samples that are positive for COVID-19 and samples that are negative for COVID-19.

### 4.7. Hardware resources and simulation environment

The allocation of robust computational resources listed in Supplementary Table S3 was pivotal in successfully developing and training our deep learning models for COVID-19 diagnosis from chest X-ray images. Utilizing high-performance hardware components, including the Intel Core i7-12700K CPU and NVIDIA GeForce RTX 3080 Ti GPU, allowed us to efficiently process vast volumes of data and perform complex matrix computations, thus expediting the training process. This strategic choice significantly reduced training times and enabled the exploration of intricate model architectures. Furthermore, the abundant 32GB of RAM and the extensive 1 TB or more SSD storage were instrumental in ensuring the seamless loading of data, preventing potential bottlenecks, and accommodating the storage needs for our extensive dataset and model checkpoints.

Complementing our powerful hardware setup, the adoption of essential image processing, statistics and machine learning, and deep learning toolboxes provided in the MATLAB 2021a are used for developing, fine-tuning, and rigorously evaluating our deep neural networks. The Windows 10 operating system further contributed to a stable and reliable research environment. This fusion of computational resources and software tools facilitated our pursuit of precise COVID-19 diagnosis and laid the foundation for transparent, accessible, and collaborative research.

### 4.8. The infected regions

Thermal maps are acquired using thermal imaging camera sensors, which play a unique role in COVID-19 diagnosis. These images record the change in body temperature, which can be very useful in the study and diagnosis of patients suffering from fever or other respiratory distress related to COVID-19. These images are overlapped with chest X-ray images to provide the radiologists with a multidimensional view, which assists in the localization of the affected region in the lungs. The fusion of thermal images with radiographic data dramatically improves the detection of COVID-19; in the case of subtle radiographic findings, it still achieves higher diagnosis accuracy. Moreover, the thermal maps assist in the ongoing monitoring of patient progress, which offers an early insight into disease treatment plans or disease deterioration, thereby assisting healthcare providers in making timely and informed decisions. When we detected COVID-19, we used an essential technique to see possibly contaminated regions—(34) work to imagine deep learning outcomes complex networks influenced this technique. We begin at the image’s top-left corner, blocking a rectangular area of MxN or a square area of dimension M rows and N columns within the X-ray sample each time to predict the occlusal image. Suppose the model wrongly classifies a picture of COVID-19 as a picture of non-COVID due to this region’s occlusion. In that case, this location will be called a likely polluted region in thoracic X-ray pictures. But if an area’s occlusion has little effect on the model’s projection, we should conclude that the region is free from contamination.

**Figure 8:**
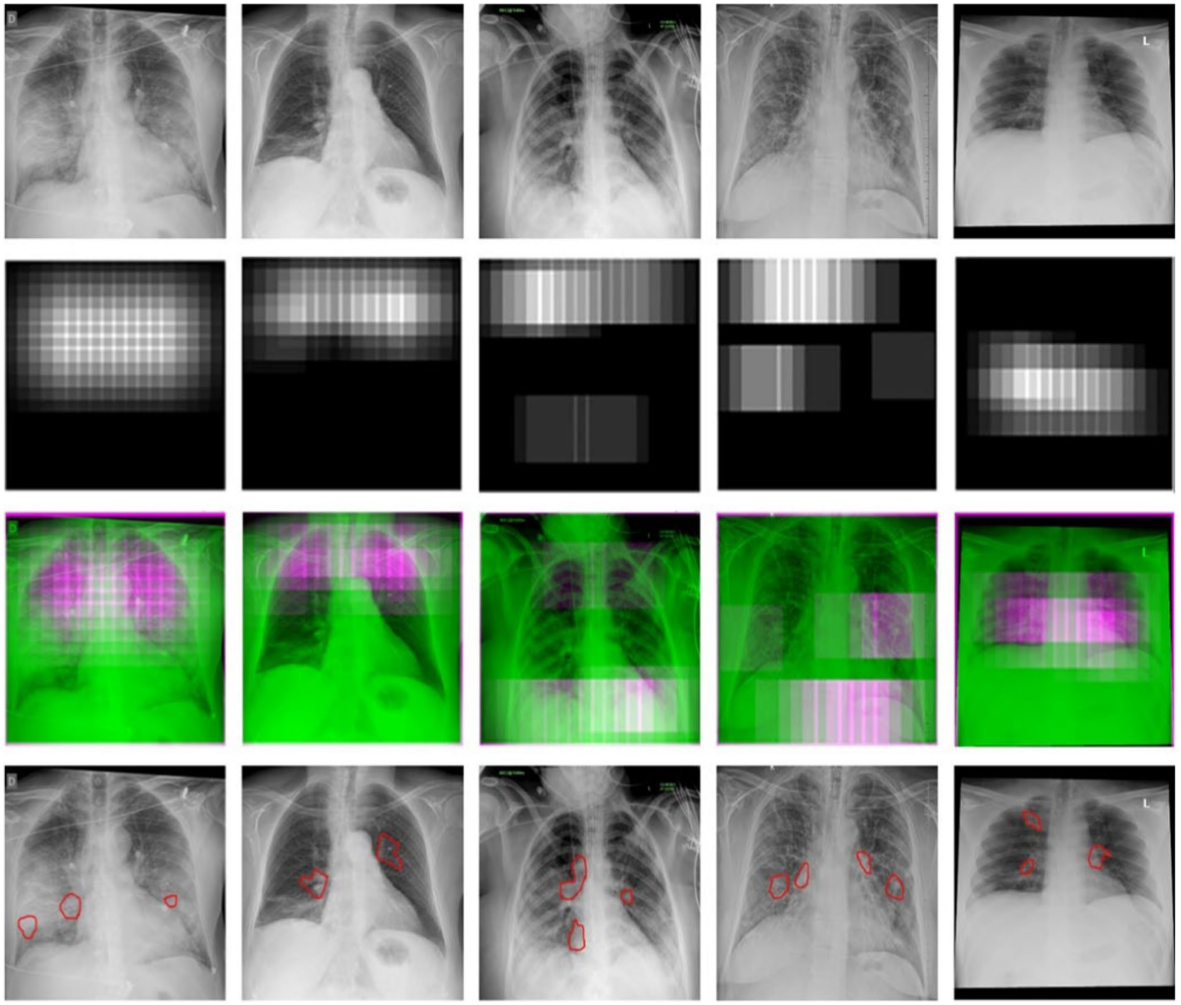
The detection of COVID-19-affected regions in the testing X-ray samples using the inception-v3 CNN model.

We can also have a sad map of infected areas detecting coronavirus by repeating this process for different slippery N x N windows and moving them each time with an S phase. Figure 8 shows the regions detected in six examples of COVID-19 photos from our test sample. In the last section, possible COVID-19 disease areas are identified and annotated in yellow color by our experts, who are certified by the Council of Radiology and Council of Medical Sciences. Regions annotated by the radiologist and experts in COVID-19 disease are in good agreement with the thermal mass produced.

## 5. Conclusion

For the sake of detecting COVID-19 and COVID-19 affected who are also diabetic, a standard dataset of 3k X-ray images is created and confirmed with the COVID-19 labels from the board-certified radiologist. The dataset is available for researchers and can be used as a benchmark dataset for COVID-19 prediction using machine-learning models. We reported that four pre-trained deep neural network models (Inception-v3, ShuffleNet, Inception-ResNet-v2, and NASNet-Large) are used to detect COVID-19 using X-ray images by fine-tuning the model’s parameters. We conducted a detailed experimental analysis on the COVID-Xray-3k dataset test set to assess these four models’ Sensitivity, specificity, ROC, and AUC performance. These models had an average specificity rate of about 90% for a sensitivity rate of 98 percent. This is encouraging because it shows promise for using X-ray images to diagnose COVID-19. This research used a set of publicly available images that included about 1,000 Normal images, 1,000 COVID-19 images, and 1,000 X-ray images of patients suffering from COVID-19 and also diabetic. The work presented here represents one of the earliest attempts at Covid-19 chest X-ray analysis and dataset preparation, which resulted in a time-sensitive correlation when the two aspects were combined. However, because there are only a few publicly available COVID-19 images, more experiments on a more extensive set of clearly labeled COVID-19 images are needed to estimate the accuracy of these models more reliably.

## Data availability statement

The original contributions presented in the study are included in the article/Supplementary material, further inquiries can be directed to the corresponding author.

## Author contributions

MS: Conceptualization, Data curation, Formal analysis, Investigation, Methodology, Software, Supervision, Validation, Visualization, Writing – original draft, Writing – review & editing. NS: Conceptualization, Data curation, Formal analysis, Investigation, Methodology, Software, Supervision, Validation, Visualization, Writing – original draft, Writing – review & editing. BS: Conceptualization, Data curation, Formal analysis, Investigation, Methodology, Software, Supervision, Validation, Visualization, Writing – original draft, Writing – review & editing. TH: Conceptualization, Data curation, Formal analysis, Investigation, Methodology, Software, Supervision, Validation, Visualization, Writing – original draft, Writing – review & editing. AA: Conceptualization, Data curation, Formal analysis, Investigation, Methodology, Software, Supervision, Validation, Visualization, Writing – original draft, Writing – review & editing. SA: Conceptualization, Data curation, Formal analysis, Investigation, Methodology, Software, Supervision, Validation, Visualization, Writing – original draft, Writing – review & editing. FA: Conceptualization, Data curation, Formal analysis, Investigation, Methodology, Software, Supervision, Validation, Visualization, Writing – original draft, Writing – review & editing.
