# Supplementary material for: Exploring transfer learning in chest radiographic images within the interplay between COVID-19 and diabetes
Source: Front Public Health. 2023 Oct 18;11:1297909. doi: 10.3389/fpubh.2023.1297909 (PMC10619728; doi:10.3389/fpubh.2023.1297909)
Supplement: Supplementary file 1 [file Data_Sheet_1.docx]

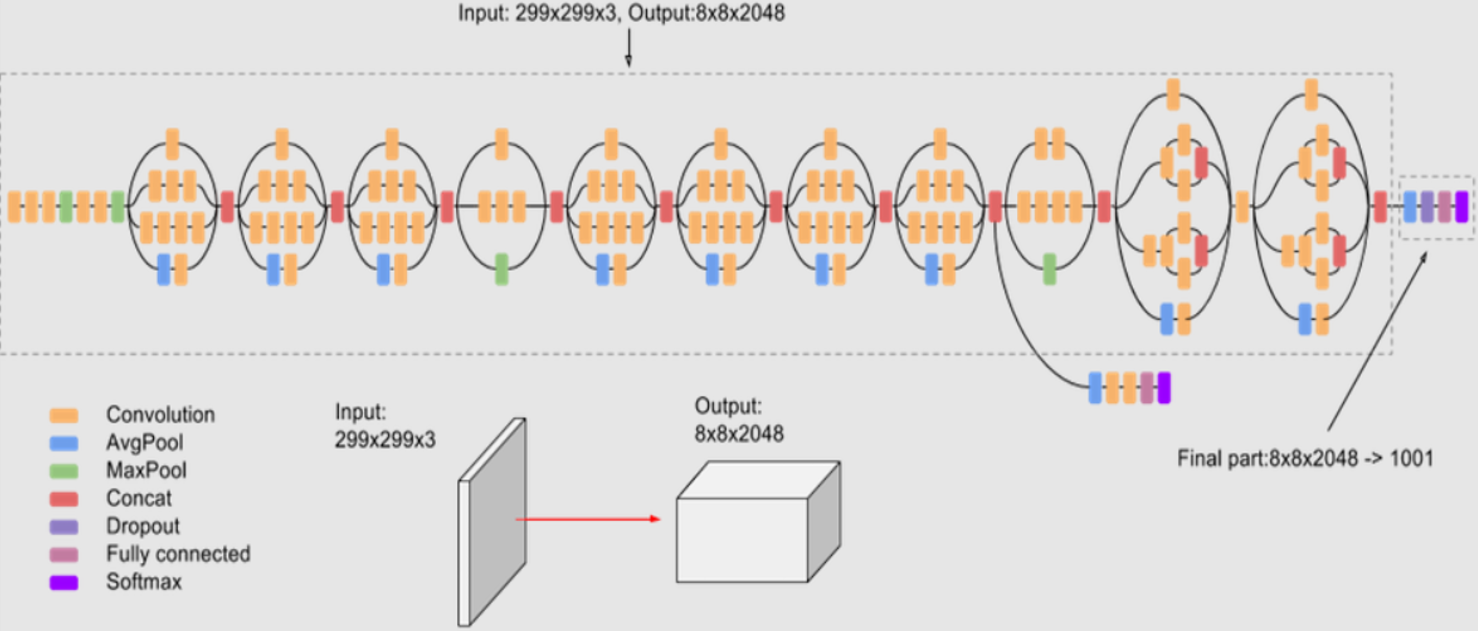


Figure 1: The Inception-v3 architecture (24).


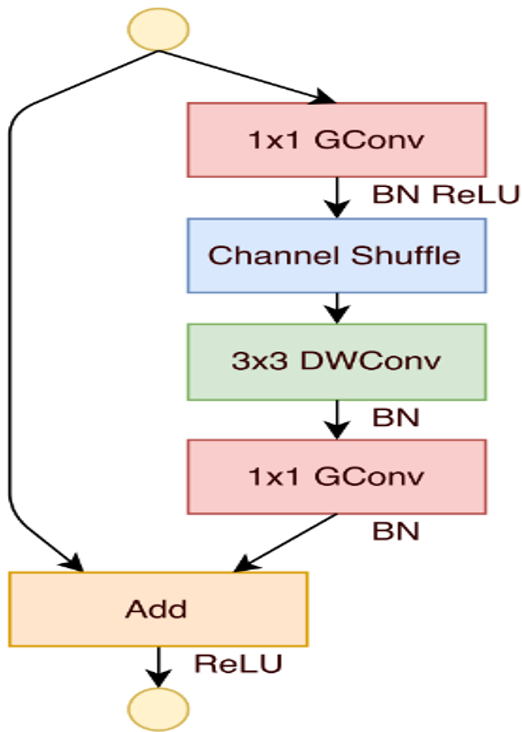


Figure 2: The structural design of ShuffleNet CNN.


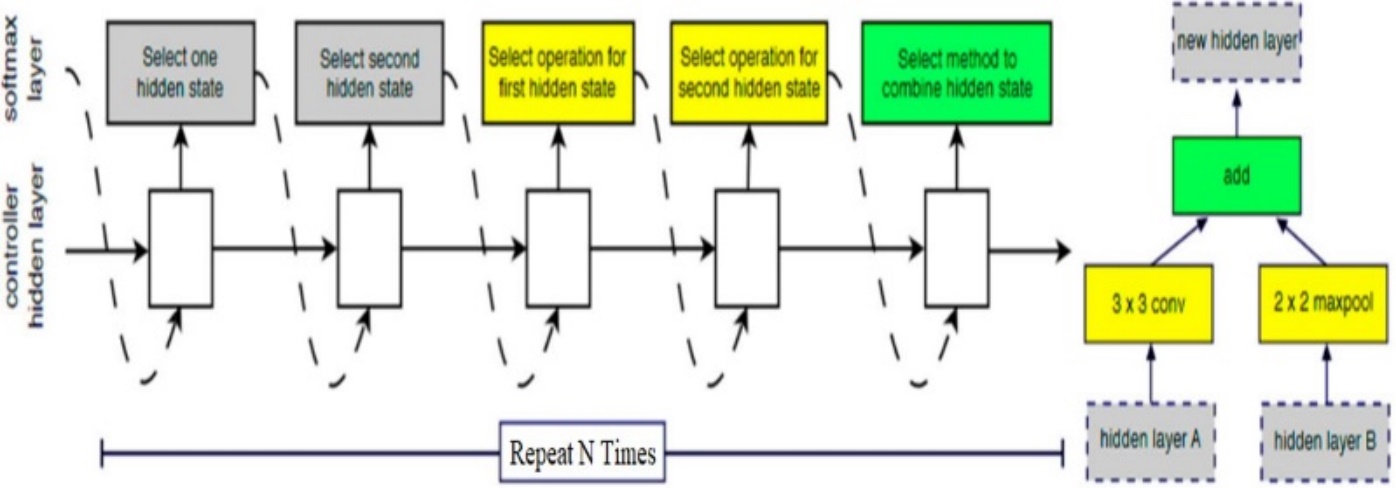


Figure 3: Shows the structural design of the NASNet-Large CNN features learning block (25).


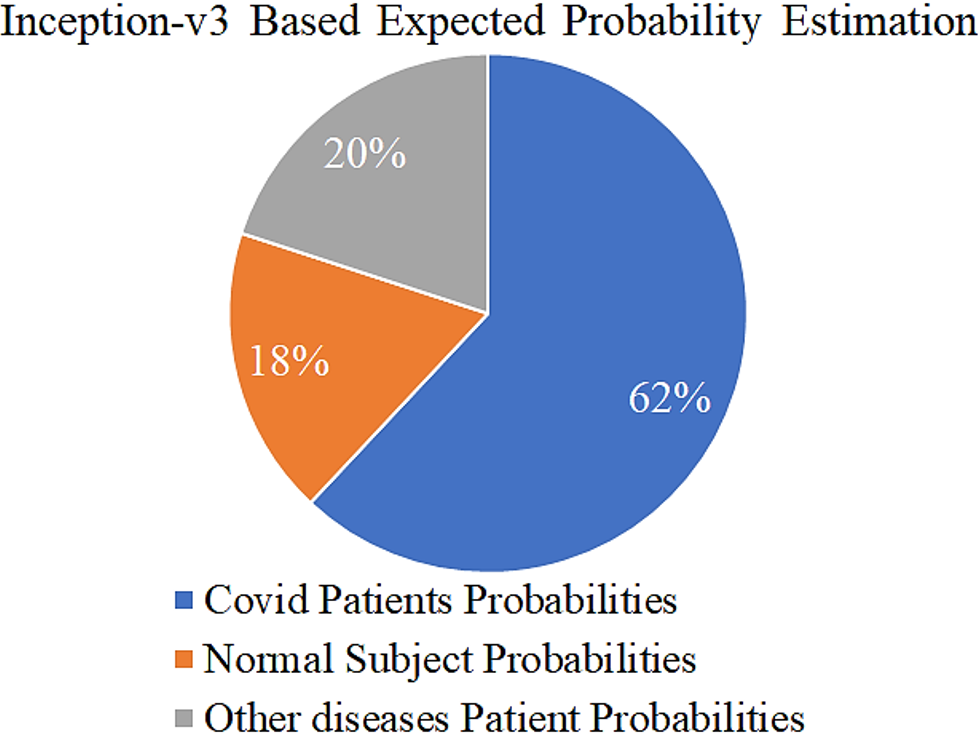


Figure 4: The probabilities estimated by the Inception-v3 CNN model using the testing samples.


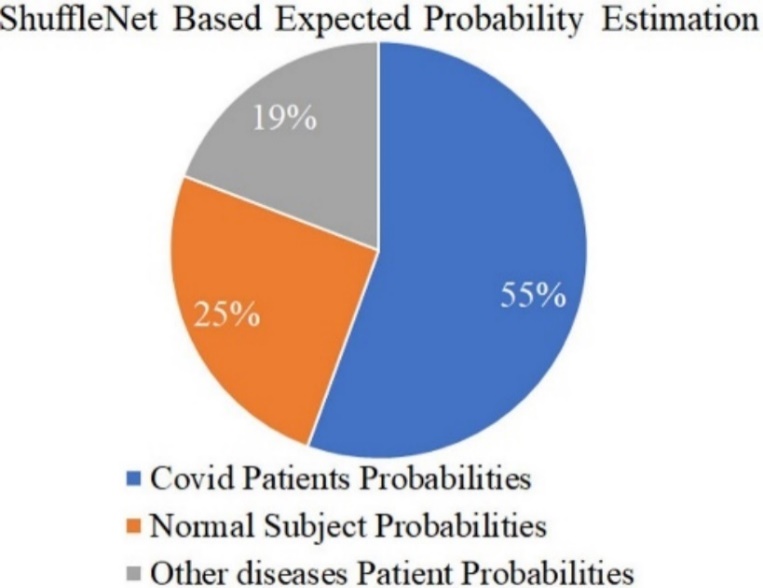


Figure 5: The probabilities estimated by the ShuffleNet CNN model using the testing samples.


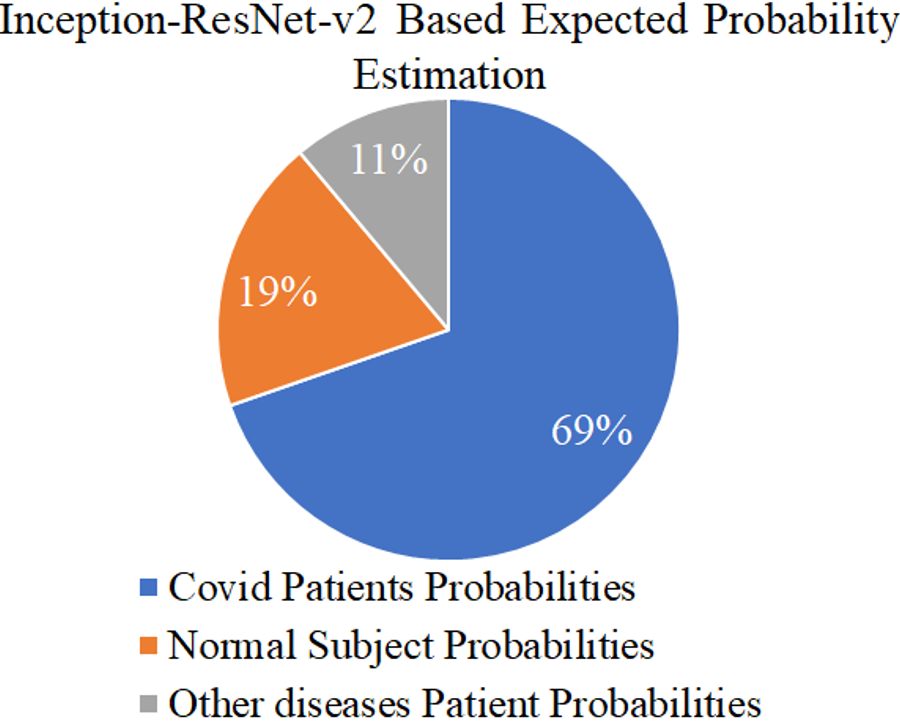


Figure 6: Shows Inception-ResNet-v2 estimated probability scores on the test sample.


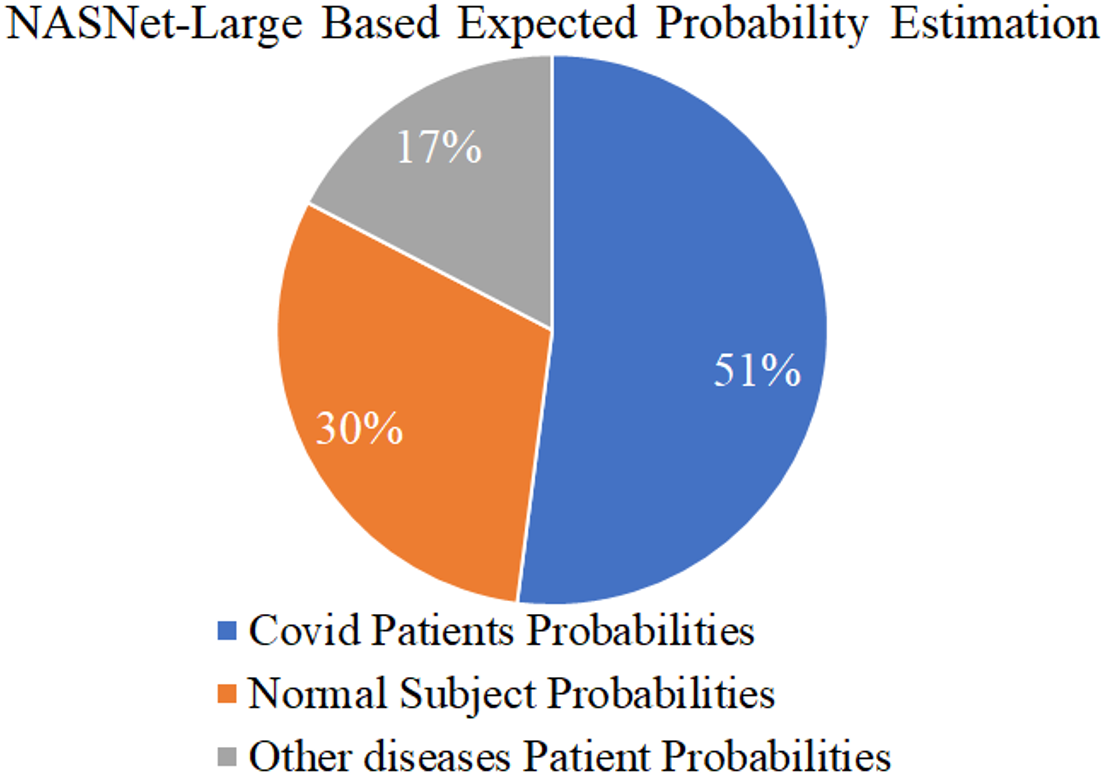


Figure 7: DesneNet-121 estimated probability scores on the test set.


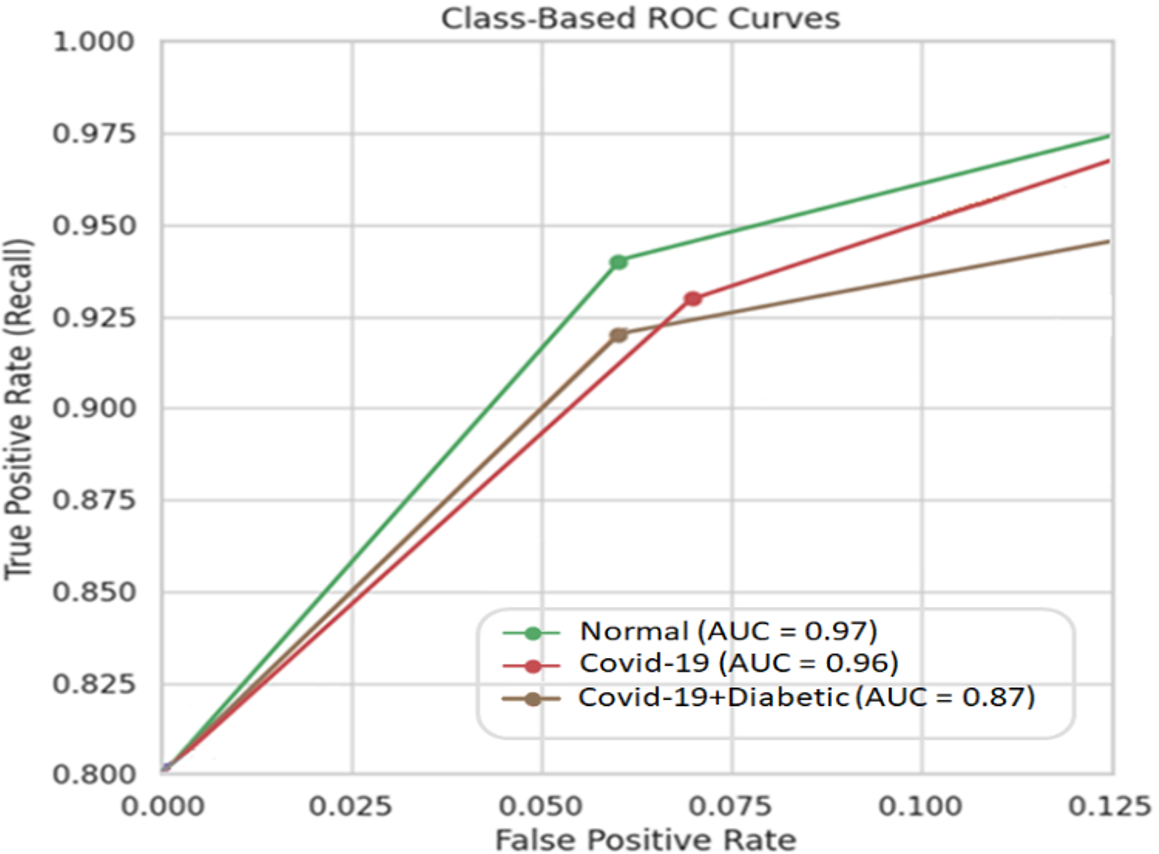


Figure 8: The ROC curve of 3 Classes on the test set.

Table 1: Hyperparameter Tuning Results and Optimal Selections.

| Hyperparameter | Value/Method | Optimal Selected |
| --- | --- | --- |
| EPOCH | 10, 20, 50 , 100 | 100 |
| Optimizer | SGDM, Adam, RMSprop | Adam |
| Shuffle Each Iteration | True, False | True |
| Learning Rate | 0.01, 0.001, 0.0001 | 0.0001 |
| Batch Size | 16, 32, 64, 128 | 64 |
| Dropout Rate | 0.2, 0.7, 0.7 | 0.5 |
| Weight Decay | 0.001, 0.0001, 0.00001 | 0.001 |
| Learning Rate Schedule | Constant, Step Decay, Adaptive | Step Decay |
| Early Stopping | 5, 10, 15 | 10 |

Table 2: Comparison of four deep neural networks.

| Models | Sensitivity | | | Specificity | | |
| --- | --- | --- | --- | --- | --- | --- |
| Inception-v3  ShuffleNet  Inception-ResNet-v2  NASNet-Large | 95%  94.2%  95.4%  91% | ±  ±  ±  ± | 2%  2%  2%  2% | 91.06%  82.32%  95.3%  90.8% | ±  ±  ±  ± | 1.9%  2%  1%  2% |

Table 3: Computational Resources Utilized for Deep Learning Model Development.

| Hardware Specification | Simulation Environment |
| --- | --- |
| CPU: Intel Core i7-12700K | Programming Language: MATLAB |
| GPU: NVIDIA GeForce RTX 3080 Ti | Simulation Environment: MATLAB 2021a |
| RAM: 32 GB DDR4 | Operating Systems: Windows 10 |
| Storage: 1 TB SSD | Supporting Toolboxes: Digital Image Processing,  Computer Vision System, Statistics & Machine Learning  and Deep Learning |
| Operating system |  |
| Machine learning framework |  |
